# Yeast prebiotics mitigate lead toxicity in Nile tilapia through physiological and ultrastructural improvements

**DOI:** 10.1038/s41598-026-37841-z

**Published:** 2026-03-05

**Authors:** Nadia A. El-Fahla, Amina A. Dessouki, Mahmoud E. Mohallal, Heba N. Gad EL-Hak, Mohamed S. Yusuf, Heba M. A. Abdelrazek, Ranwa A. Elrayess

**Affiliations:** 1 Department of Zoology, Faculty of Sciences, Suez Canal University, Ismailia, Egypt; 2 Department of Pathology, Faculty of Veterinary Medicine, Suez Canal University, Ismailia, Egypt; 3 Department of Nutrition and Clinical Nutrition, Faculty of Veterinary Medicine, Suez Canal University, Ismailia, Egypt; 4 Faculty of Veterinary Medicine, King Salman International University, South Sinai, Egypt; 5 Department of Physiology, Faculty of Veterinary Medicine, Suez Canal University, Ismailia, Egypt

**Keywords:** Heavy metals, Mannan oligosaccharides, Β-glucan, Nile tilapia, Hepatotoxicity, Bioaccumulation, Aquatic pollution, Biochemistry, Environmental sciences, Physiology, Zoology

## Abstract

Lead (Pb) contamination is a major threat to aquaculture and food safety, mainly due to its hepatotoxic and oxidative impacts on fish. This study evaluated the ameliorative role of yeast-derived prebiotics, mannan oligosaccharides (MOS) and β-glucan (βG), against Pb-induced toxicity in Nile tilapia (*Oreochromis niloticus*). A total of 180 fish were allocated into four treatment groups for eight weeks: control, MOS/βG-supplemented diet (0.3%), Pb exposure (10 mg/L), and combined Pb with MOS/βG supplementation. Pb exposure significantly reduced serum total protein, albumin, and globulin while increasing hepatic enzymes, indicating liver dysfunction. Severe hepatic necrosis, hepatopancreatic degeneration, gill lamellar fusion, and mitochondrial disruption were also observed, alongside elevated Pb residues in liver > gills > muscles. Dietary MOS/βG markedly restored altered biochemical markers, improved histoarchitectural and ultrastructural integrity, and reduced Pb accumulation in all tissues compared with Pb-exposed fish (*p* < 0.05). These findings demonstrate that MOS/βG supplementation enhances physiological resilience to heavy metal stress and is associated with reduced Pb bioaccumulation, highlighting their potential as a sustainable nutritional intervention to protect fish health and reduce tissue contamination in Pb-polluted aquaculture environments.

**Supplementary Information:**

The online version contains supplementary material available at 10.1038/s41598-026-37841-z.

## Introduction

Aquaculture has a crucial role in meeting global protein demand, supporting livelihoods, and advancing sustainable food systems. However, heavy metal contamination mainly from industrial discharge, agricultural runoff, and aquaculture intensification remains a major bottleneck affecting production efficiency and fish health^1^. Nile tilapia (*Oreochromis niloticus*) is one of the world’s most widely cultured species due to its rapid growth and tolerance to diverse environmental conditions. Yet, its position in the aquatic food chain increases the likelihood of heavy metal bioaccumulation, creating potential public health hazards through consumption of contaminated fish^2^.

Among hazardous metals, Pb is a persistent and highly toxic pollutant that induces oxidative stress, metabolic disturbances, and immunotoxicity in fish^3^. The liver and gills are primary Pb targets due to their key roles in metabolism, detoxification, and ion regulation. Histopathological and ultrastructural alterations in these organs are considered sensitive biomarkers of heavy metal toxicity^4^. Increasing Pb residues in edible fish tissues, such as muscle, have exceeded permissible limits set by international health authorities, raising concern for food safety^5^.

Yeast-derived prebiotics, particularly MOS/ΒG have emerged as promising functional feed additives in aquaculture due to their multifaceted biological activities^6^. MOS, derived from the outer cell wall of *Saccharomyces cerevisiae*, consists primarily of mannan-rich polysaccharide fractions that can selectively bind pathogenic bacteria and mycotoxins in the gastrointestinal tract, thereby reducing their colonization and absorption^7^. β-glucans, specifically β-1,3/1,6-glucan polymers from yeast cell walls, are recognized by pattern recognition receptors including dectin-1 and complement receptor 3 on immune cells, triggering downstream immunomodulatory cascades that enhance phagocytic activity, respiratory burst, and cytokine production^8^. Beyond their immunostimulatory effects, both MOS and β-glucan improve intestinal integrity by promoting tight junction protein expression, enhancing villus height and goblet cell density, and supporting beneficial microbiota colonization, which collectively strengthen gut barrier function and nutrient absorption efficiency^9^. Notably, these yeast derivatives possess metal-binding capacity through their hydroxyl and carboxyl functional groups, enabling them to chelate divalent heavy metals such as Pb²⁺, Cd²⁺, and Cu²⁺ in the digestive tract, thereby reducing their bioavailability and systemic absorption^10^. Furthermore, MOS and β-glucan have been shown to upregulate endogenous antioxidant defense systems, including superoxide dismutase, catalase, and glutathione peroxidase, which mitigate oxidative stress induced by environmental pollutants^11^. Recently, functional feed additives have gained attention as eco-friendly strategies to reduce pollutant-induced toxicity in fish. Yeast-derived prebiotics, including MOS/βG, have been shown to improve immune competence, gut health, and antioxidant defenses in aquaculture species^12^. Previous studies of Xue et al.^11^ have separately reported that MOS/βG can mitigate hepatic enzyme disturbances and enhance detoxification capacity under stress conditions. However, the combined protective efficacy of MOS/βG against Pb toxicity has not yet been fully elucidated, particularly in relation to cellular ultra-structure alterations through transmission electron microscopy. This research aimed to determine whether supplementation with 0.3% MOS/βG can alleviate biochemical impairments, tissue injury, and cellular degeneration as well as reduce Pb accumulation in liver, gills, and muscle of O. niloticus exposed to waterborne Pb under environmentally relevant conditions. The findings support functional feed strategies that contribute to healthier aquaculture systems and improved food safety in metal-polluted regions.

## Materials and methods

### Fish rearing and experimental conditions

A total of 180 healthy juvenile Nile tilapia (35.43 ± 0.36 g; 8 weeks old) were obtained from a private fish farm and transported to the laboratory. Fish were treated in a 4% NaCl bath for 10 min to remove ectoparasites. After sex determination, fish were randomly distributed at a density of 15 fish per 60 L glass aquarium (60 × 30 × 40 cm^3^), previously disinfected with NaCl solution.

Water quality parameters were monitored throughout the trial. Temperature was maintained at 26–28 °C using heaters, pH averaged 7.56, and dissolved oxygen remained close to saturation with continuous aeration. Ammonia concentration: NH_3_/NH₄⁺ remained below 0.02 mg/L and dissolved oxygen: 6.8 ± 0.4 mg/L throughout the experiment. A semi-static system was applied, where water siphoning and renewal were performed three times per week with re-adjustment of Pb concentration when applicable. Natural photo period conditions were maintained. Fish were acclimated for four weeks prior to experimentation and monitored daily for clinical health.

### Experimental design and diets

Fish were randomly assigned into four groups, each comprising three replicates (*n* = 45 per group; 15 fish per replicate aquarium): Control: Basal diet with no supplementation. The basal diet formula contains Fish meal (25%), soybean meal (30%), corn gluten (15%), wheat bran (20%), corn starch (5%), fish oil (3%) and vitamin-mineral premix (2%). MOS/βG: Diet supplemented with 0.3% Beta-MOS^®^ (mannan oligosaccharides and β-glucan; EURO MARK, Italy). The product contains approximately 20% MOS and 15% β-glucan, with the remainder being other yeast cell wall components. Beta-MOS^®^ powder (0.3%) was thoroughly mixed with finely ground basal diet ingredients using a mechanical mixer (Hobart mixer, USA) for 20 min to ensure homogeneous distribution. The mixture was then pelleted using a laboratory pellet mill with addition of water (~ 15%), and pellets were air-dried at room temperature for 24 h. Pellets were stored in sealed plastic bags at 4 °C until use. Batch homogeneity was confirmed by analyzing three random samples from each batch for consistent supplementation levels. Pb-exposed: 10 mg L^−1^ Pb acetate (Sigma-Aldrich, Germany) dissolved in water + basal diet. The Pb concentration (10 mg L^−1^) represents a high but sub-lethal challenge dose relevant to heavily contaminated aquaculture systems receiving agricultural drainage and industrial effluents in Egypt and other developing regions, where Pb has been detected at 6–12 mg L^−1^ in surface waters^13^. While not representative of typical farm conditions, this concentration has been shown to induce measurable sub-lethal toxicity in Nile tilapia while maintaining acceptable survival for physiological assessments^14^, making it suitable for evaluating protective interventions under worst-case pollution scenarios. Pb + MOS/βG: 10 mg L^−1^ Pb acetate in water + 0.3% Beta-MOS^®^ diet. The 0.3% inclusion level of MOS + βG was chosen based on prior studies of El-Fahla et al.^15^. Higher doses have shown limited incremental benefits and may impair palatability, whereas lower doses lead to inconsistent biological responses. Preliminary in-house feeding trials also confirmed that 0.3% supported good growth and health outcomes while maintaining uniform feed intake across treatments. Fish survival was monitored daily, and the overall survival rate was calculated at the end of the experiment. Diets were formulated following the nutritional requirements of growing tilapia according to Jobling^16^ ensure equal and adequate nutrient levels across treatments, providing ~ 29% crude protein and 4100 kcal kg^−1^ gross energy. Ingredients were finely milled (175-µm mesh), mixed, pelleted with water, and stored at 4 °C. Fish were fed daily at 3% of body weight divided into two equal meals (morning and afternoon), adjusted weekly based on weight measurements for 8 weeks. Mortality was recorded daily. Fish survival was monitored daily throughout the eight-weeks experimental period. Dead fish were immediately removed, count, and recorded by treatment group and replicated aquarium to track temporal mortality patterns. At the conclusion of the experiment, cumulative survival rates were calculated for each treatment group as the percentage of fish alive relative to the initial stocking density (*n* = 45 fish per group, with three replicate aquaria of 15 fish each).

Water Pb concentration was monitored weekly in each aquarium using atomic flame absorption spectrophotometry (FAAS). Measured Pb concentrations ranged from 9.2 to 10.8 mg L^−1^ (mean 9.8 ± 0.6 mg L^−1^) throughout the experiment. At each water renewal (three times weekly), Pb concentration was re-adjusted to 10 mg L^−1^ by adding calculated amounts of Pb acetate stock solution to maintain consistent exposure.

### Ethical approval 

All procedures were approved by the Animal Ethics Committee, Faculty of Veterinary Medicine, Suez Canal University (Approval No. 2019035). This study is reported in accordance with the ARRIVE guidelines. All experimental procedures, animal handling, and sampling methods were performed in accordance with relevant institutional, national, and international guidelines and regulations.

### Sampling procedures

#### Blood sampling and serum biochemical analysis

At the end of the experiment, blood was collected from the caudal vein of anaesthetized fish of 15 fish per group (5 fish randomly selected per aquarium × 3 aquaria) (clove oil/ethanol 1:10) following Perdikaris et al.^17^. Serum was separated by centrifugation (3000 rpm, 15 min) and stored at − 70 °C for biochemical assays (total protein, albumin, globulin, ALT, AST, ALP). Total protein, albumin, and liver enzyme activities (ALT, AST, ALP) were quantified using commercial diagnostic kits (Biodiagnostic, Egypt) following the manufacturer’s protocols (Gornall et al., 1949; Reitman & Frankel, 1957; Belfield & Goldberg, 1971). Total protein concentration was measured using the Biuret method (Cat. No. TP 2500), while serum albumin was determined by the Bromocresol green colorimetric method (Cat. No. AL 1030). Globulin concentration was calculated by subtracting albumin from total protein. Hepatic enzyme activities were assessed using kinetic and colorimetric assays: alanine aminotransferase (ALT, Cat. No. AL 1100) and aspartate aminotransferase (AST, Cat. No. AS 1200) were measured via the kinetic UV method, while alkaline phosphatase (ALP, Cat. No. AP 1300) activity was determined using the colorimetric method described by Belfield and Goldberg (1971). All assays were performed in duplicate following the manufacturer’s instructions, and absorbance readings were obtained using a spectrophotometer at the wavelengths specified for each parameter to ensure accurate quantification of serum protein and enzyme markers. Globulin was calculated as: Globulin = Total protein − Albumin.

### Tissue sampling and histopathological examination

Nine fish per group (3 fish per aquarium × 3 aquaria) were dissected, and liver weight was recorded for hepatosomatic index calculation. Liver and gill tissues were rinsed. Portions were fixed in Bouin’s solution for histopathology and in 3% glutaraldehyde (0.1 M sodium cacodylate buffer, pH 7.2) for transmission electron microscopy (TEM). For Pb analysis, muscle, liver, and gill samples were stored at − 20 °C. Representative samples of liver and gill tissues (~ 5 mm^3^) were excised and immediately fixed in Bouin’s fluid at a tissue-to-fixative volume ratio of 1:20 for 48 h to ensure complete fixation. The tissues were then washed under running tap water to remove picric acid residues and processed through a graded ethanol dehydration series (70%, 80%, 90%, 95%, and 100%; 20 min in each step). Following dehydration, samples were cleared through two changes of xylene (30 min each) and infiltrated in molten paraffin wax at 60 °C with two changes of soft paraffin for 1 h each to facilitate full saturation. Tissues were subsequently embedded in hard paraffin blocks to provide optimal sectioning stability. Thin tissue section (5 μm thickness) were prepared using a rotary microtome, mounted on glass slides, and stained with hematoxylin followed by eosin (H&E) (Avwioro, 2010). A total of nine specimens per tissue per treatment were examined using a Leitz Diaplan light microscope (Leitz, Germany), and photomicrographs were digitally captured to document pathological alterations.

### Transmission electron microscopy

Small fragments (~ 1 mm^3^) of liver and gill tissues of 6 fish per group (2 fish per aquarium × 3 aquaria) were immediately excised and immersed in 3% glutaraldehyde prepared in 0.1 M sodium cacodylate buffer (pH 7.2) at a fixative-to-tissue volume ratio of 20:1, ensuring adequate penetration. Primary fixation was conducted for 24 h at 4 °C. Samples were then rinsed three times in the same buffer and post-fixed in 1% osmium tetroxide for 2 h at 4 °C. Following fixation, tissues were dehydrated through ascending ethanol concentrations (50% → 100%) and transitioned to propylene oxide before embedding in an Epon–Araldite epoxy resin mixture. Polymerization was performed at 60 °C for 48 h to ensure complete resin curing. Semi-thin Sect. (0.5–1 μm) were cut with a glass knife, stained with toluidine blue, and examined to select regions of interest. Ultrathin Sects. (50–100 nm) were then cut using a diamond knife on a Leica EM UC6 ultramicrotome (Leica Co., Austria), mounted on copper grids, and stained with 2% uranyl acetate followed by lead citrate (Sato et al., 2008). Sections were examined using a JEOL JEM-1011 transmission electron microscope operated at 80 kV at the Electron Microscope Unit, Assiut University.

### Pb bioaccumulation

Pb residues in muscle, liver, and gill tissues of 9 fish per group (3 fish per aquarium × 3 aquaria) were quantified using (FAAS) following the standardized digestion and analytical procedures of Beutler et al.^18^. Approximately 1.0 g of each homogenized tissue sample was weighed into acid-washed digestion tubes, mixed with 10 mL of concentrated nitric acid (HNO_3_, 65%), and pre-digested overnight at room temperature. Samples were then heated at 120 °C on a hot plate until complete digestion occurred and a clear solution was obtained. After cooling, 2 mL of hydrogen peroxide was added to ensure full oxidation of organic matter, followed by gentle heating until colorless. Digested samples were then diluted to 25 mL with deionized water and filtered prior to instrumental determination. Calibration curves were constructed using Pb standard solutions prepared from certified stock standards (Merck, Germany). Instrument performance was validated through procedural blanks, standard spiking, and duplicate analyses. Certified reference materials (CRM; fish muscle tissue, Sigma-Aldrich QC Standards) were used for accuracy control, producing spike recovery values between 92 and 105%, within acceptable limits for trace metal quantification. The method detection limit for Pb was 0.01 mg kg^−1^. All metal concentrations are expressed as mg kg^−1^ wet weight. FAAS measurements were performed using a System 45AA spectrophotometer (International Equipment Trading Ltd., USA) equipped with a Pb hollow cathode lamp and operated under manufacturer-recommended conditions. The bioaccumulation factor equation calculated as BAF = C_tissue / C_water (mg kg^−1^ tissue / mg L^−1^ water).

### Statistical analysis

Data normality was assessed u sing the Shapiro-Wilk test, and homogeneity of variances was tested using Levene’s test. All data met the assumptions for parametric analysis (*p* > 0.05). Survival data were analyzed using the Chi-square (χ²) test to determine whether mortality rates differed significantly among treatment groups, with statistical significance set at *p* < 0.05. All statistical analyses were performed using SPSS software version 20.0 (IBM Corp., Armonk, NY, USA). Means ± SE were compared using Tukey’s multiple range test with significance set at *p* < 0.05. To avoid pseudo-replication, aquarium means were used as the experimental unit for all statistical analyses (*n* = 3 replica aquaria per treatment).

## Results

### Fish survival

Fish survival was monitored daily throughout the eight-week experimental period, and cumulative mortality rates were calculated at the conclusion of the trial. Both the control group and MOS/βG-supplemented group maintained 100% survival (45/45 fish in each group) with no mortalities recorded during the entire experimental period. In contrast, the Pb-exposed group exhibited a survival rate of 91.1% (41/45 fish), with four mortalities occurring during weeks 6–8 of the exposure period, likely reflecting chronic stress and physiological impairment induced by prolonged lead intoxication. The Pb + MOS/βG co-treatment group demonstrated an improved survival rate of 97.8% (44/45 fish), with only one mortality recorded in week 7, suggesting a protective effect of dietary prebiotic supplementation against Pb-induced mortality. Statistical analysis using the Chi-square test revealed that survival differences among treatment groups were not statistically significant (χ^2^ = 4.87, df = 3, *p* = 0.18), though the Pb-exposed group showed a numerical trend toward increased mortality compared to all other groups. The reduction in mortality from four deaths in the Pb-only group to one death in the Pb + MOS/βG group represents a 75% improvement in survival under metal stress conditions.

### Serum biochemical parameters

Pb-exposed group fed basal diet significantly altered serum protein profiles and hepatic enzyme activities (one-way ANOVA, *p* < 0.05). Total protein, albumin, and globulin concentrations were significantly reduced in the Pb-exposed group compared to control fed basal diet and MOS/βG-supplemented group (*F*_3,176_ = 45.32, *p* < 0.001). MOS/βG-supplemented group significantly elevated these serum proteins relative to controls, and co-administration with Pb restored total protein, globulin, and albumin levels compared to Pb-exposed group fed basal diet (Table 1). Hepatic enzymes ALT, AST, and ALP were significantly elevated in Pb-exposed fish fed basal diet (*F*_3,176_ = 68.47, *p* < 0.001 for ALT; *F*_3,176_ = 52.19, *p* < 0.001 for AST; *F*_3,176_ = 89.64, *p* < 0.001 for ALP), indicating hepatocellular damage and membrane permeability disruption. MOS/βG-supplemented group significantly reduced enzyme activities below control levels, and co-treatment markedly attenuated Pb-induced enzyme elevation (Table 1).

**Table 1:** Influence of dietary Beta-MOS^®^ on serum biochemical parameters in lead (Pb) intoxicated nile tilapia.

Group parameters	Control	0.3% Beta-MOS^®^	Pb	Pb+Beta MOS^®^
Total protein (g/dL)	1.41 ± 0.06 ^b^	1.88 ± 0.07 ^a^	1.00 ± 0.04 ^c^	1.59 ± 0.06 ^b^
Albumin (g/dL)	0.94 ± 0.08^a^	1.06 ± 0.08 ^a^	0.56 ± 0.05 ^b^	0.93 ± 0.05^a^
Globulin (g/dL)	0.50 ± 0.04 ^b^	0.79 ± 0.08 ^a^	0.44 ± 0.04 ^c^	0.66 ± 0.08 ^a^
ALT (U/L)	19.29 ± 1.50 ^bc^	17.11 ± 1.35 ^c^	43.68 ± 3.68 ^a^	27.91 ± 0.83 ^b^
AST (U/L)	22.31 ± 1.13 ^b^	20.06 ± 1.83 ^c^	35.36 ± 1.28 ^a^	22.96 ± 1.05^b^
ALP (U/L)	38.31 ± 2.23 ^b^	19.70 ± 1.07 ^c^	67.36 ± 2.08 ^a^	35.54 ± 2.10^b^

Data represented as mean ± SE of *n* = 9 fish per group. Values denoted by different letters (a, b, & c) within the same row indicate that their corresponding means are significantly different at *p* < 0.05, as determined by one-way ANOVA followed by Tukey multiple range test. ALT: alanine aminotransferase, AST: aspartate aminotransferase, ALP: alkaline phosphatase.

### Histopathological examination in Nile tilapia liver and gills

#### Gills

**Fig. 1:**
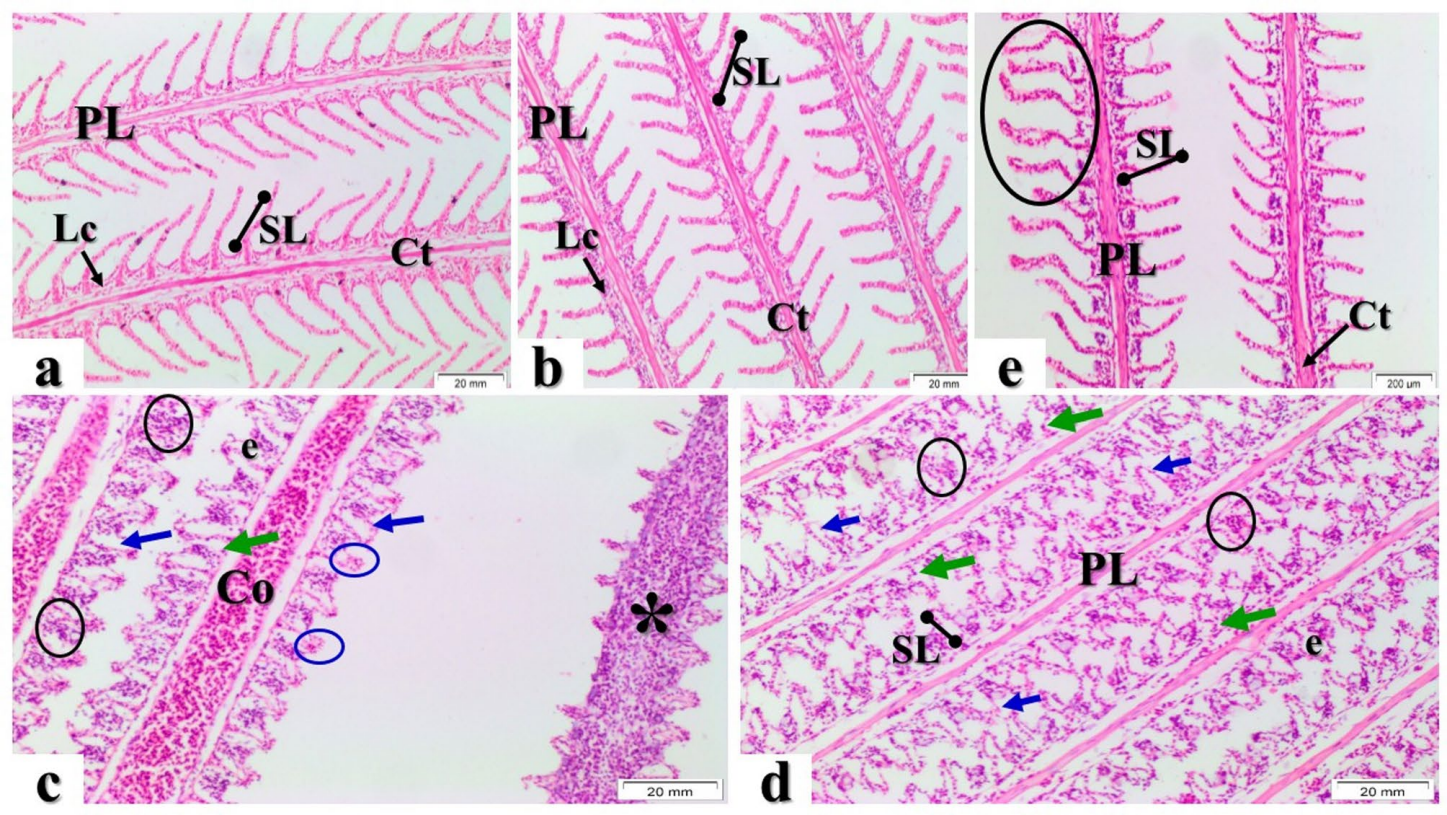
Longitudinal gill sections from control fed basal diet and MOS/βG-supplemented group (**a**) and (**b**) respectively, showed normal primary lamellae (PL) supported by cartilaginous matrix (Ct) and inter-lamellar cells (Lc) and normal secondary lamellae (SL). Sections from Pb-exposed group fed basal diet demonstrated degeneration (green arrows) of epithelial cells, complete atrophy and lamellar deformations characterized by epithelial lifting (blue arrows) associated with edema (e), lymphocytes infiltrations (black circles) and aneurisms (blue circles). In addition, PL showed congested (Co) blood vessel, and almost all PL revealed complete necrosis (asterisk) associated with lymphocytes infiltrations. (**d**) Gills sections from Pb + MOS/βG group showed an improvement of gill tissue characterized by absence of severe alterations and the SL revealed hypertrophy with slight congestion (circles). H&E stain (X 200).

The gills of both the control group control fed basal diet (Fig. 1a) and MOS/βG-supplemented group (Fig. 1b) exhibited normal primary lamellae covered by simple squamous epithelium, along with afferent, efferent arterioles, or other blood vessels. The primary lamellae contained a central cartilaginous core surrounded externally by inter-lamellar cells. Similarly, the surfaces of the secondary lamellae were lined with epithelial pavement cells; within this layer, blood sinuses containing erythrocytes could be observed separated by pillar cells.

In contrast, Pb-exposed group fed basal diet showed significant pathological changes in the gill tissues (Fig. 1c, e). Most Pb-exposed group fed basal diet displayed marked hyperplasia of inter-lamellar epithelial cells that extended into secondary lamellae resulting in partial fusion between adjacent structures. Additionally, it was noted that there were hypertrophy and curling deformities affecting secondary lamellae. All samples examined from Pb-intoxicated groups evidenced severe alterations including degeneration of epithelial layers associated with edema formation as well as congestion within their vascular components manifested through aneurysm development accompanied by lymphocytic infiltration. Furthermore, congestion was evident among blood vessels supplying primary filaments and widespread necrosis involving both types. Lamina commonly occurred alongside pronounced lymphocytes presence across most sections examined.

In those Pb + MOS/βG group showed a variable reduction in histopathological lesions emerged; substantial improvements included an absence or clear mitigation regarding features such as epithelial hyperplasia, fusion or necrosis linked to inflammatory cell infiltrates and aneurysms seen previously without supplementation (Fig. 1d).

#### Hepatopancreas

**Fig. 2:**
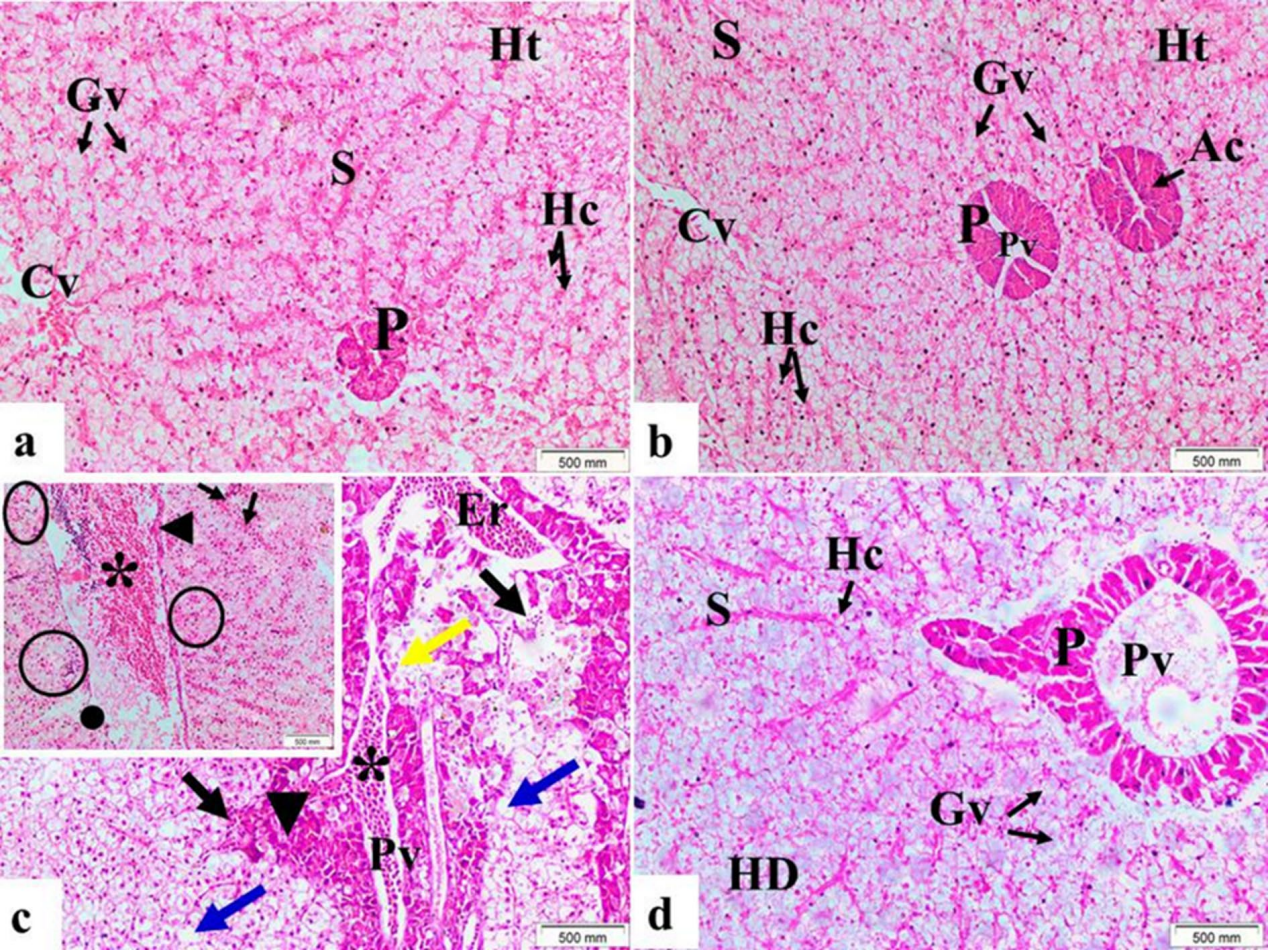
Liver tissue sections from control fed basal diet and MOS/βG-supplemented group (**a**) and (**b**), respectively showed hepatic tissue (Ht) with normal hepatocytes (Hc), glycogen vacuoles (Gv), sinusoids (S) and central vein with erythrocytes. Also, the pancreas (P) showed normal acinar cell (Ac) surrounding portal vein (Pv). (**c**) Section of liver from Pb-exposed fed basal diet showed loss of normal histological structure between hepatic and pancreatic tissues characterized by; degeneration of pancreatic tissue, atrophied acinar cells (yellow arrow), focal necrosis (▼) of associated with lymphocytic infiltrations and hemorrhage (black arrows). Dilation and congestion (asterisk) of Pv with erythrocytes (Er) and vacuoles were clearly visible (blue arrows) in Ht. (**c**) Window showed dilation and congestion (asterisk) in hepatic vessel, the endothelial linings of hepatic vessel were thickened (◄) and other side showed hemolysis (●), hemorrhage with lymphocytic infiltrations (circles) and congestion of sinusoids (black arrows) were observed in the hepatic tissues. H&E stain (X 200). (**d**) Liver section from Pb + MOS/βG group demonstrated significant improvement of hepatic and pancreatic tissues, normal hepatocytes (Hc), glycogen vacuoles (Gv) and sinusoids (S) and pancreas (P) with normal acinar cell (Ac) surrounding portal vein (Pv). Parts of hepatic tissue showed cloudy, cellular swelling or hydropic degeneration (HD). H&E stain (X 200).

The hepatopancreas from both the control group fed basal diet (Fig. 2a) and MOS/βG-supplemented group (Fig. 2b) exhibited normal histological features without any alterations after eight weeks of experimentation. In these samples, hepatic tissue displayed polygonal-shaped hepatocytes, which were not arranged in distinct cords or lobules but had clearly visible cell membranes and large eccentric nuclei. Glycogen vacuoles were evident within the hepatocytes. Furthermore, hepatic sinusoids irregularly distributed among the hepatocytes formed an extensive network lined by simple squamous endothelial cells possessing elongated dark nuclei. The exocrine pancreatic acinar cells appeared scattered throughout this region surrounded by clusters of hepatocytes adjacent to portal areas.

Pb-exposed group fed basal diet showed several pronounced histopathological lesions within the hepatopancreas. Marked findings included hydropic degeneration and cytoplasmic vacuolization within many hepatocytes; dilation along with blood congestion containing erythrocytes was noted in hepatic veins and sinusoids. Necrosis often co-occurs with lymphocytic infiltration plus hemorrhage across various regions of affected livers (Fig. 2c). Some examined tissues also demonstrated mild thickening or occasional hemolysis of vascular endothelium lining certain vessels observed under microscopy analysis relative to controls. Most sections of pancreatic tissue revealed degenerative changes marked by disorganization between typical glandular architectures at interfaces adjoining hepatic components with numerous damaged and atrophied acinar structures and associated bleeding into surrounding parenchyma. Portal vein segments routinely showed significant congestion composed predominantly from red blood cell accumulation interspersed directly amid leukocytes presence while other venous channels elsewhere displayed abnormal dilation patterns post-Pb exposure periods showed in the examined sections.

However, Pb + MOS/βG group showed improvement ensued concerning morphology compared side-by-side those Pb-exposed group fed basal diet. Overall microscopic architecture neared resemblance toward healthy control organs once more albeit some residual cloudy swelling, hydropic change persisted (Fig. 2d).

### Transmission electron microscopy (TEM)

#### Gills ultrastructure

**Fig. 3:**
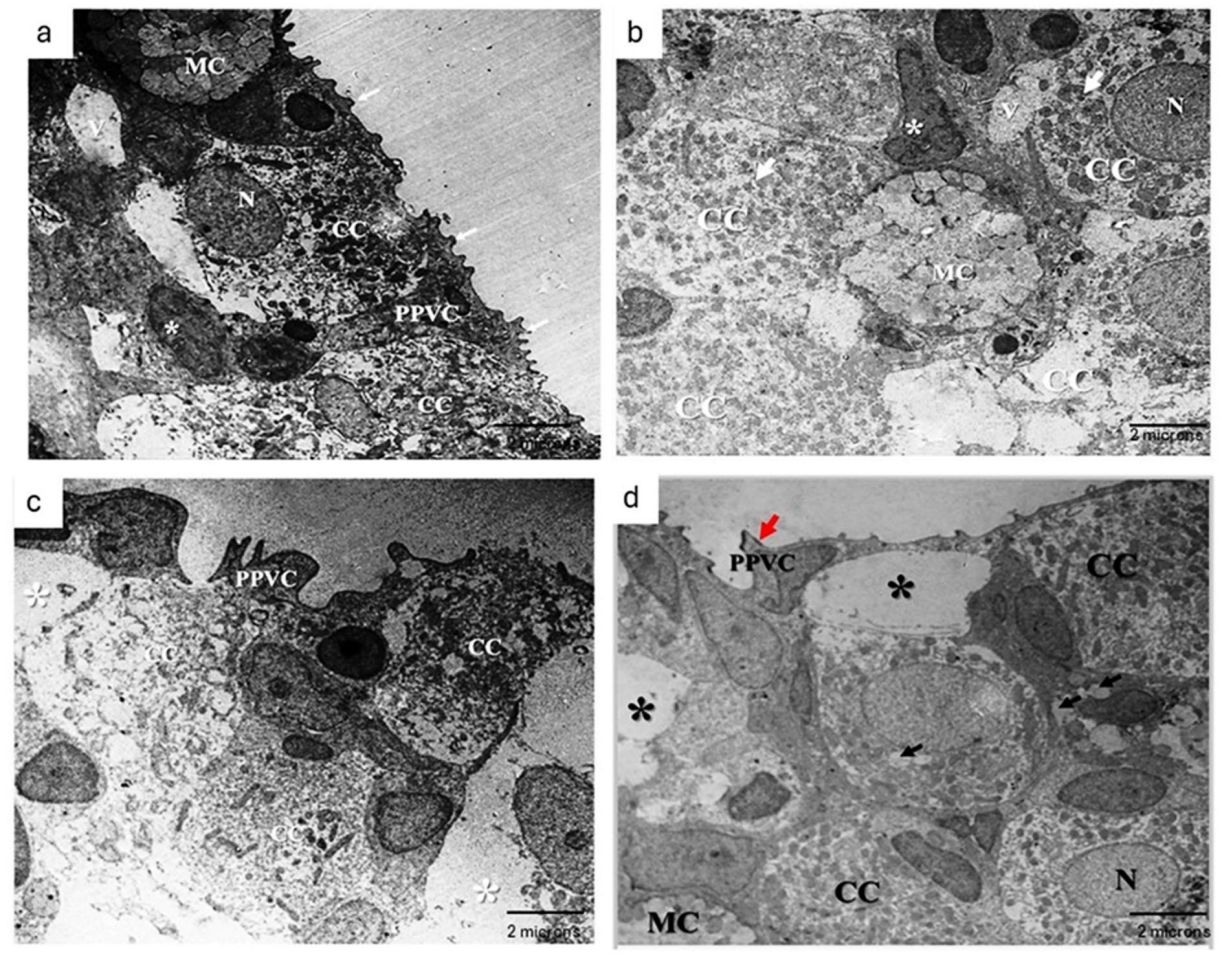
TEM photomicrograph of gills from (**a**) control fish fed basal diet revealed the superficial region of primary lamellae. The epithelium of PL showed chloride cells (CC) with abundant mitochondria and well-defined nucleus, mucous cell (MC) containing pale mucous secretory vesicles and primary pavement cells (PPVC) with several micro-ridges (arrows) were covered the CC. Note, undifferentiated cells (asterisk) found in the superficial region between adjacent CC. (**b**) MOS/βG-supplemented group revealed the superficial region of primary lamellae. The epithelium of PL showed hyper-activation of chloride cells (CC) with rich mitochondria and well-defined nucleus, mucous cell (MC) containing pale mucous secretory vesicles. Note, undifferentiated cells (asterisk) found in the superficial region between adjacent CC. (**c**) Pb-exposed fed basal diet showed the superficial region of primary lamellae (PL) with a variety of ultrastructural alterations. Atrophy of chloride cells (CC) with severe disruption and loss of mitochondria, loss of micro-ridges of primary pavement cells (PPVC) along with widening intercellular spaces (white asterisks). (**d**) Pb + MOS/βG group showed chloride cells (CC) with abundant mitochondria and well-defined nucleus (N), vacuoles (black arrows) within the cells, mild widening of intercellular spaces (asterisks), loss of micro-ridges (red arrow) of PPVC and mucous cell (MC).

The gills of control group fed basal diet (Fig. 3a) have primary lamellae partitioned longitudinally by a central vascular axis, and mostly four kinds of cells were recognized in the primary epithelium. The first cell is chloride cells, which are characterized by rich mitochondria and frequently dispersed in the superficial layer of epithelium, beside the mucous cells with dense and pale mucous secretory vesicles and lipid droplets. The third cell of primary lamellae is the pavement epithelial cell (PVC), which is seen covering the epithelium of lamellae and bearing regular finger-like projections called micro-ridges. The basal lamina is the deep layer, which is lined by the fourth type, which is called basal cells. In addition to a network of undifferentiated cells found between the adjacent basal cells. In the secondary epithelium, the pillar cells are found in the middle of secondary lamellae, enclosing the blood spaces with thin flanges that extend to the neighboring pillar cell, and red blood cells are observed inside the blood spaces. In addition, the PVC of the secondary lamellae are polygonal in shape, less dense and display smooth surface with lower number and long micro-ridges than PVC of primary lamellae. In addition, the gills chloride cells of MOS and βG supplemented Nile tilapia (Fig. 3b) were characterized by hyperplasia with abundant mitochondria.

TEM studies in the gills of Pb-exposed group fed basal diet (Fig. 3c) showed a variety of ultrastructure alterations. The most prominent abnormalities detected in the superficial layer of primary epithelium were severe intercellular spaces among epithelial cells, marked degeneration of chloride cells, a few chloride cells showed large cytoplasmic vacuoles, while another cell showed signs of necrosis associated with abnormal or deformed nuclei and severe loss of mitochondria. PPVC appeared electronically dense and lost their numerous micro-ridges. Hyperplasia and hypertrophy of mucous cells were also seen within the primary epithelium. In the secondary lamellae, marked interstitial fluid infiltration or edema associated with severe detachment or lifting of squamous epithelium of PVC were the most pathological signs that clearly observed. Besides these, vasodilation of blood vessels with marked congestion and presence of mild intercellular spaces among secondary epitheliums. At certain places, the pillar cells showed loss of their structural features, and they rarely enclosed blood spaces.

However, the electronic micrographs of Pb + MOS/βG group (Fig. 3d) showed some remaining lesions like these of Pb group, but TEM studies revealed marked improvement in primary lamellae. No signs of necrosis or degeneration of chloride cells were observed in all examined sections, in addition the mucous cells showed normal appearance without hypertrophy or hyperplasia. Besides, the secondary lamellae showed mild alterations without completely recovering.

#### Hepatopancreas ultrastructure

**Fig. 4:**
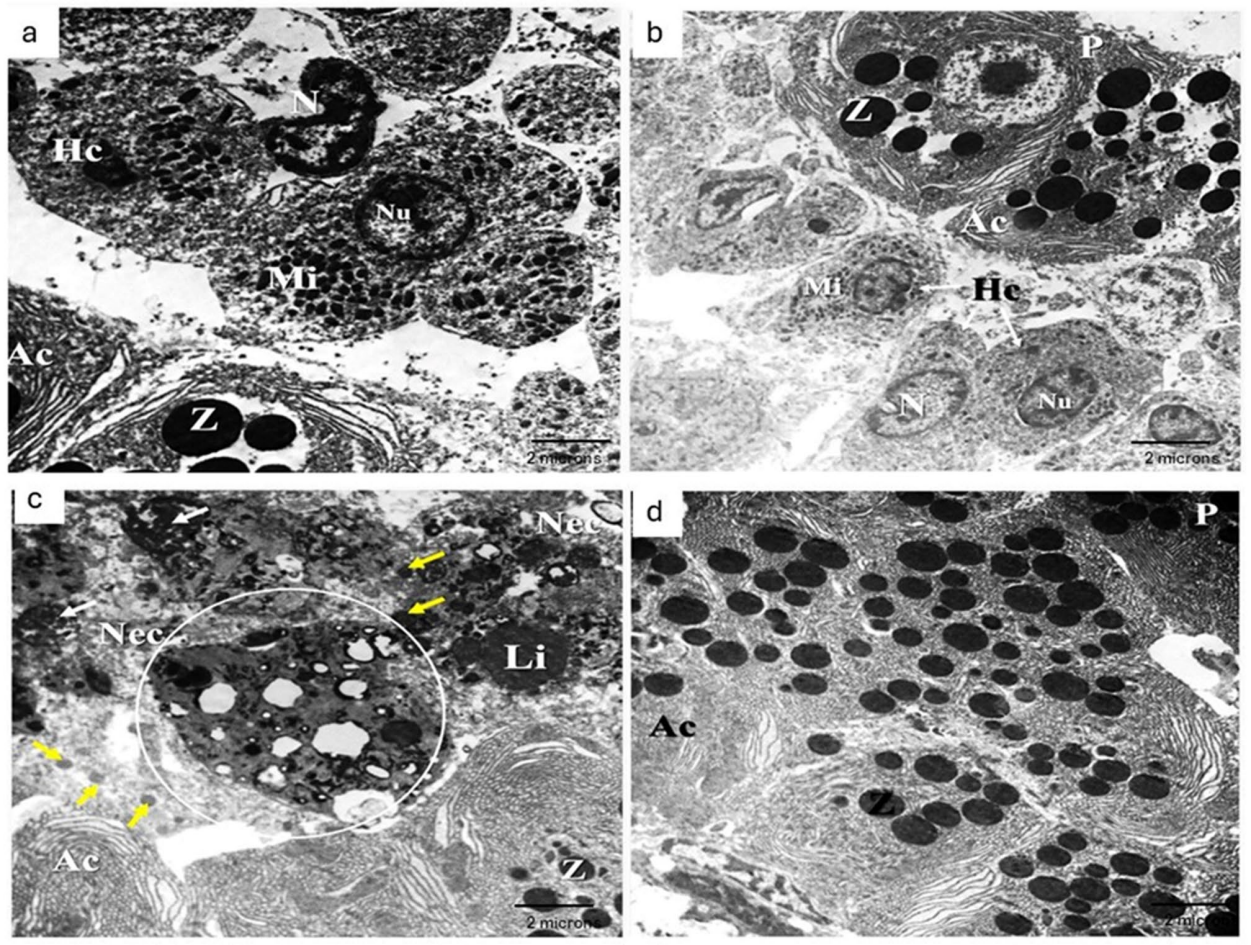
TEM photomicrograph of hepatopancreas from (**a**) control fed basal diet showed hepatocytes (Hc) with well-defined nucleus (N) and nucleolus with abundant mitochondria (Mi). The cytoplasm of hepatocytes rich in glycogen granules were observed. As long pancreas (P) with acinar cells (Ac) included zymogen granules (Z). (**b**) MOS/βG-supplemented group showed hepatocytes (Hc) with well-defined nucleus (N) and nucleolus and abundant mitochondria (Mi), observe that the cytoplasm of hepatocytes rich in glycogen granules. Besides pancreas (P) with acinar cells (Ac) included zymogen granules (Z). (**c**) Pb- fed basal diet revealed severe lesions within liver parenchyma included cellular necrosis (Nec), hyperplasia of peroxisomes (yellow arrows), nuclear deformities characterized by augmentation of nucleolus with marked expansion of heterochromatin (white arrows) and accumulation of lipid (Li) droplets. (**d**) Pb + MOS/βG group revealed electronically normal appearance of pancreatic tissue (P) with normal acinar cells (Ac) include zymogen granules (Z).

The hepatopancreas of control group fed basal diet (Fig. 4a) and MOS/βG-supplemented group (Fig. 4b) composed of hepatic parenchyma and exocrine pancreatic cells. Ultrastructural, the hepatic parenchyma was made of polygonal hepatocytes characterized by a single round nucleus that usually located central to peripheral and attached with parallel stacked cisternae of rough endoplasmic reticulum. The nuclei of hepatocytes contained abundant euchromatin, a little peripheral heterochromatin as well as scattered clumps of heterochromatin surrounding the nucleolus. The cytoplasm showed different organelles, which included mitochondria, lysosomes, and peroxisomes. There were abundant storage materials, mostly glycogen granules that take the rosette shape appearance. These granules coalesced together to occupy a large area of cytoplasm. Mitochondria appeared elongated or oval and were nearly located to the nucleus as well as between the cisternae of rough endoplasmic reticulum. Peroxisomes and lysosomes scarcely appeared and randomly distributed in the cytoplasm. Few lipid droplets observed scattered in the cytoplasm, were round and small. The exocrine acinar cells are distinguished from the other cell types by the presence of secretory granules named zymogen granules and a well-developed rough endoplasmic reticulum. The zymogen granules were usually abundant and located at the apical portion of the exocrine cells. The nuclei of the pancreatic cells were generally like those of hepatocytes but located at the basal portion of the cell. The rough endoplasmic reticulum revealed dilated cisternae that concentrically distributed and occupied almost the area of the acinar cell.

The hepatocytes of Pb-exposed group fed basal diet (Fig. 4c) presented distinct alterations. Most hepatocytes showed cellular necrosis, proliferation of peroxisomes, and accumulation of large lipid droplets with a marked increase in number. Additionally, nuclear abnormalities characterized by some hepatocytes revealed deformations of the nuclear membrane or giant nucleus with fragmented nucleolus, atrophy of the nucleus, and expansion of heterochromatin. Secondary lysosomes included heterogeneous components showed an extensive increase in size and number within the cytoplasm of hepatocytes. In all TEM-examined hepatocytes, the cytoplasm revealed depletion of glycogen granules and mitochondria as well as loss of rough endoplasmic reticulum cisternae. Few examined TEM hepatocytes revealed myelin bodies. Besides, the acinar cells also revealed necrosis, atrophy, and nuclear deformations.

Pb + MOS/βG group (Fig. 4d) showed variables decrease of ultrastructure lesions with marked improvement in both hepatic and pancreatic tissue characterized by absence of necrosis, nuclear deformations, cellular organelles loss, and few appearances of peroxisomes and lysosomes.

### Pb bioaccumulation residues

Pb residues in muscle, liver, and gill tissues were significantly elevated in Pb-exposed fish fed basal diet compared to all other groups (*F*_3,32_ = 78.23, *p* < 0.001 for muscle; *F*_3,32_ = 124.56, *p* < 0.001 for gills; *F*_3,32_ = 156.89, *p* < 0.001 for liver). Pb + MOS/βG group significantly reduced Pb accumulation in all examined tissues compared to the Pb group fed basal diet (*p* < 0.05), with liver exhibiting the highest bioaccumulation followed by gills and muscle (Table 2). MOS/βG supplementation significantly reduced muscle Pb accumulation by 42%, residues remained above regulatory limits (FAO/WHO, 0.5 mg kg^−1^). Dietary MOS/βG supplementation significantly reduced Pb bioaccumulation across all examined tissues compared to the Pb-only group (*p* < 0.05). Specifically, the Pb + MOS/βG co-treatment group demonstrated a 42% reduction in muscle Pb residues (1.90 ± 0.26 mg/g), a 35% reduction in gill Pb content (9.03 ± 0.28 mg/g), and a 38% reduction in hepatic Pb accumulation (23.71 ± 2.43 mg/g) relative to Pb-exposed fish receiving the basal diet alone.

**Table 2:** Effect of dietary Beta-MOS^®^ on Pb bioaccumulation in some selected vital organs of lead (Pb) intoxicated nile tilapia.

Group parameters	Control	0.3% Beta-MOS^®^	Pb L-1	Pb L-1 + Beta-MOS^®^
Muscles (mg kg^−1^ wet weight) muscle Pb residues to the FAO/EU maximum permissible limit (0.5 mg kg^−1^)	0.153 ± 0.08 ^c^ (below limit)	0.03 ± 0.01^c^ (below limit)	3.54 ± 0.25 ^a^ (exceeds limit by 4.3×)	1.90 ± 0.26 ^b^ (still exceeds limit by 2.5×, but 42% reduction vs. Pb-only)
Gills (mg kg^−1^ wet weight)	6.73 ± 0.34 ^c^	2.24 ± 0.21 ^c^	32.57 ± 4.16 ^a^	9.03 ± 0.28 ^b^
Liver (mg kg^−1^ wet weight)	13.40 ± 0.59 ^c^	10.00 ± 0.51 ^c^	58.12 ± 3.62 ^a^	23.71 ± 2.43 ^b^

Data represented as mean ± SE of *n* = 9 fish per group (3 fish per aquarium × 3 aquaria) for each tissue type. Values denoted by different letters (a, b, c) within the same row indicate that their corresponding means are significantly different at *p* < 0.05, as determined by one-way ANOVA followed by Tukey multiple range test.

## Discussion

Pb metal in water systems represents a significant global environmental pollutant issue. These issues Pb to detrimental influences on fish and carry risks to human health via the food chain^19–21^. Pb can readily enter the environment and subsequently infiltrate the bloodstreams of humans and animals, affecting various organs including gills, intestines, kidneys, brain, liver, bones, and gonads^22,23^. There is a growing demand for environmentally friendly feed additives as alternatives to antibiotics and synthetic chemicals, aimed at reducing the harmful effects of pollutants particularly heavy metals on farmed fish^24–26^. So far, an effective treatment for Pb toxicity in aquaculture has not been established^23^. Prebiotics are increasingly being adopted as immunostimulants in aquaculture worldwide, largely due to their cost-effectiveness, accessibility, ease of formulation, and low toxicity to both humans and aquatic species^27^. This study examined the potential protective effects of prebiotics βG and MOS (Beta-MOS^®^) on Pb-induced toxicity in Nile tilapia.

Liver constitutes a vital part in detoxifying xenobiotic, and it considered the main target organ affected by heavy metals^28^. The Liver enzymes ALP, AST, and, ALT are critical indicators that reflected the extent of liver alterations, and the elevation of their activities indicated the unhealthy status of fish^29^. Therefore, the activities of ALT, AST, and ALP were evaluated to determine the magnitude of Pb induced toxicity in hepatic tissues. In the herein results, exposure to Pb increased the ALT, AST and ALP activities in the Sera of Nile Tilapia. These changes may result from Pb-induced oxidative load induction that caused cellular and pathological alterations to liver tissues. The elevated activities of ALT, ALP, and AST enzymes in blood sera are attributable to mitochondrial degradation, which arises from oxidative stress in hepatic tissue and muscle injury^30^. The latter resulted in liberation of ALT, ALP and AST from hepatocytes into blood and elevating their levels. Similar results were obtained by Alm-Eldeen et al.^31^, Osman et al.^32^ and Giri et al.^23^. The Pb induced hepatic lesions were reflected directly on hepatic function and protein synthetic capacity. Whereas Pb exposure produced marked reduction in globulin, total protein, and albumin serum contents. The liver is considered the main source of albumin however, globulin is produced from other tissues beside liver^33^. Albumin and globulin, the primary serum proteins, are essential for maintaining osmotic pressure and are involved in transporting exogenous chemicals, endogenous metabolites, and immune components that help protect against infections^34^. Albumin is the principal plasma protein, confirmed to play a crucial role in keeping osmotic pressure and counteracting the effects of drugs, toxic metals, and other chemicals in blood^35^. The existence of globulin in blood is considered an optimistic sign of immunity and health^36–38^. Serum total protein acts as a comprehensive biomarker, encompassing protective enzymes, metabolites present in vital bodily fluid, and stress hormones; thus, it is a vital indicator of the humoral immune system^39^. Alterations or fluctuations in total protein levels in sera are principally resulted from plasma volume alterations, therefore any stressful condition that produces such condition may amend the total protein levels^40^. Total protein serves as an indicator of protein metabolism; diminished levels may be observed in hepatic disorders^41^.

The administration of MOS and βG to Pb-intoxicated fish ameliorated the serum liver enzymes activity. Therefore, hepatic lesions were restored near to control one. All these effects positively influenced the hepatic synthetic power of albumin, globulin and total protein that were improved. Similar results were got by Ayyat et al.^42^.

The histopathological observations are good biomarkers reflecting the inflammatory alterations caused by heavy metals exposure in the aquatic animals in response to environmental contaminants^43^. Also, the histopathological sections reflected the robust relationship between the harshness of tissue damage and the accumulated metal levels in tissues. Toxins initially entered the fish body via skin, gills and/or intestines, after that they were absorbed and deposited in the cells causing severe alterations including inflammation and lipid peroxidation^44^. Current histopathological alterations in Pb-exposed Nile tilapia revealed that Pb induced several histopathological deteriorations in liver and gills. Under metal exposure conditions, most of the fish tissues showed severe alterations and inflammation^44,45^.

Gills are the primary target organ in fish, being to direct contact with different unfavorable environmental conditions. In the existed histological investigation, the gills of control and MOS and βG group fish showed normal structural organization of the lamellae. However, the gills of Pb-exposed fish showed several histological alterations, after 8 weeks of Pb-exposure period. The marked detected lesions of gill toxicity were hyperplasia, hypertrophy and degeneration of epithelial cells, epithelial lifting associated with edema, lamellar fusion, and aneurisms on secondary lamellae as well as curling of lamellae. In addition, necrosis of gill filaments, lymphocytic infiltrations, hemorrhage and congestion were also observed. The later results were in concurred with previous records, which reported the same severe alterations in Pb-exposed gills of blue spotted ray *Dasyatis kuhlii*^45^, *Capoeta capoeta*^46^, common carp *Cyprinus carpio*^47^ and Nile tilapia^48^. Gills alterations including hyperplasia and hypertrophy of the epithelial cells, epithelial lifting, besides partial fusion of some secondary lamellae are types of defense mechanisms against toxicant to reach the bloodstream. These alterations served as a barrier against contaminants entry via increased the distance between epithelium and waterborne pollutants^49,50^. Mohamed^51^ reported degeneration in cells of the gill filaments caused due to oxygen lack because of gill intoxication. Edema is one of the more frequent lesions observed in gill epithelium of fish exposed to heavy metals whereas, fluid accumulation in gill tissues leads to epithelia lifting^50^. Aneurisms have been detected as an outcome of necrosis in the pillar cell and designated as a sturdy response in gills against toxicants including Pb^52^. Other study showed that necrosis of gill filaments associated with lymphocytic infiltrations causing gill dysfunction and reflected continuous absorption of Pb into the gill tissues^48^.

In current study, administration of MOS and βG to Pb group revealed marked improvement of gills tissues than Pb group. These results were concurred with Petit, Wiegertjes^53^ and Dawood et al.^54^ who reported that dietary βG was immunostimulant and anti-oxidant agent which could eliminate the gill pathological toxicity caused by Pb. The later effect could be explained by the reduction of Pb accumulation induced by MOS and βG that reduced the gills’ retrogressive changes. The reduction in Pb bioaccumulation observed in MOS/βG-supplemented Nile tilapia is likely mediated through several complementary mechanisms acting along the gut–gill axis. Enhanced intestinal morphology and tighter epithelial junctions limit paracellular Pb entry from the lumen, thereby decreasing systemic uptake at the first barrier level^55^. In parallel, mannan and β-glucan possess metal-binding and adsorptive properties that can sequester divalent cations in the gut contents, lowering the fraction of free Pb available for absorption^56^. The well-documented immunostimulant and antioxidant effects of yeast-derived prebiotics also help preserve the structural integrity of gill and intestinal epithelia by attenuating oxidative injury, which maintains selective permeability and prevents non-specific metal influx^57^. Additionally, although not directly quantified in the present work, previous studies indicate that yeast components may upregulate intracellular metal-binding proteins such as metallothioneins, providing an intracellular buffering system that favors Pb sequestration and detoxification rather than its redistribution to edible tissues^58^.

The fish liver acts as a primary site for detoxification mechanisms^59^. The screening of liver histopathological alterations in fish is a marked accurate and sensitive way to explore the toxic potentials of xenobiotic molecules in aquaculture^49^. Hepatopancreatic sections from both the control group and MOS/βG-supplemented group exhibited normal histological architecture. However, the hepatopancreatic sections in all Pb-exposed fish revealed several histopathological alterations, after 8 weeks of toxicity. The liver with Pb revealed signs of hepatocytes’ vacuolar degeneration, dilatation and blood congestion of hepatic vein and sinusoids with erythrocytes, degeneration of pancreatic tissue, lymphocytic infiltration and hemorrhage in the hepatic tissue. Besides, loss of hepatocytes’ contacts and atrophy of acinar cells and hemolysis in blood vessels. These remarks were reinforced by the rise of liver enzymes and Pb residues in Pb-exposed tissues. The hepatic degenerative alterations and necrosis in the Pb-intoxicated liver might reflect the acquisitive influence of Pb in liver tissues. Fish accumulated higher levels of metals in liver than gills or other tissues^60^. These hepatic lesions caused probably owing to the liver role in excretion and metabolism of toxic xenobiotic^28^. Similar results were noticed previously in different fish species intoxicated with Pb; blue spotted ray^45^, common carp^61^ and Nile tilapia^62^. The present results were in agreement with Mobarak, Sharaf^63^ who observed that silver sailfin molly (*Poecilia latipinna*) exposed to 0.8 mg /L Pb revealed several liver lesions. Their lesions included atrophy in hepatocytes, dilatation and congestion of sinusoids and central veins, besides loss of normal structure of the hepatocytes.

Treatment of Pb group with MOS and βG revealed marked improvement in hepatopancreatic tissues than Pb group, which might be due to the antioxidative influence of dietary MOS and βG. These results were concurred with Dawood et al.^54^ who reported that dietary βG had antioxidant effect which could eliminate the toxic effect of Pb on hepatopancreatic tissues. Hassaan et al.^64^ reported that Nile tilapia fed diet supplemented with the extract of backer’s yeast improved the hepatocytes’ structure, which directed that the yeast derivatives could enhance liver functions and progress tissues restoration.

Transmission electron microscopic observations revealed several ultrastructural alterations in Pb-exposed gills. The abnormalities detected in the primary epithelium were marked lesions in CCs and mucous cells. Chloride cells are the most active cells in gills, often they are target of heavy metals toxicity owing to their main role in ions absorption^65^. Hypertrophy, necrosis and depletion of mitochondria of CCs were observed, these lesions have been reported previously in different fresh water fishes after Pb-exposure^66^. The CCs were degenerated even if, underneath a threshold of metal exposure, they remain performing their activity. However, in the highest metal concentration, the severity of ultrastructural alterations increased due to reduction in the expression of Na^+^/K^+^ ATPase^67^. The decline of Na^+^/K^+^ ATPase activity were detected in freshwater fishes’ gills after Pb exposure^68^. In current study, the ultrastructure appearance of Pb-exposed gills showed loss of numerous micro-ridges of PPVC, besides hyperplasia of MCs. These changes cause increase of mucous secretion that have been recorded in freshwaters fishes after Pb-exposure^3^. The main functions of PVCs micro ridges were increasing the surface area of ion absorption and involved in retention of mucus, which considered the main protective response of gills against environmental contaminants^69^. Also, secondary lamellae of gills in Pb-exposed fish showed structure-less of pillar cell and epithelial lifting associated with edema. Similar cytopathological lesions were evidenced previously and proved to be signs of defense mechanisms against pollutants, as well as these lesions affect the gill function^70^. These alterations might interfere with normal respiratory function and prime disruption of fish health circumstances causing high mortality rate as observed in the herein study.

The supplementation of MOS and βG to the Pb-exposed group ameliorated Pb deteriorating changes. This positive effect might be correlated with immunostimulant and antioxidant effects of βG and MOS as confirmed previously by Selim, Reda^71^.

The hepatopancreas ultrastructural of the Pb-exposed group showed depletion of cytoplasmic organelles in hepatocytes as glycogen granules, mitochondria, and rough endoplasmic reticulum cisternae. These toxic effects were formerly detected in Pb-exposed fish and assumed to be unspecific responses of liver cells due to Pb-induced hepatic stress^72^. The diminution of glycogen, in the herein study has also been illustrated in different fish species exposed to environmental pollutants^73^. This depletion might be correlated with disruption of protein synthesis, as well as unuse of lipids for protein-lipid conjugation. The proliferation of secondary lysosomes, perixosomes, and autophagic vesicles, as well as the appearance of myelinic bodies in the hepatocytes, were stated previously in fish intoxicated with Pb, Hg, and Cd^72^ concurred with the present observations. He declared that Pb caused nuclear deformaties, changes of heterochromatin and the nuclear envelope and varaitions of nucleoli, together with destruction of RER. These lesions caused by Pb induced oxidative stress that caused impairment to nucleic acid and protein metabolism^74^. The anomalous lipid accumulation (steatosis) in Pb-intoxicated liver was considered a pre-necrotic stage and has been noticed in fish suffering from heavy metals toxicity^75^. Steatosis is a condition of lipid accumulation within hepatocytes caused by a disorder in lipid metabolism that leads to a reduction of fish growth performance, feed utilization, immunity, and stress resistance^76^. Necrosis in the present study had been proved as an irreversible injury in tissue structure and function, which reflected impairment of enzyme activities, interference in protein synthesis and lipid utilization, and association with oxidative stress^77^. The addition of MOS and βG to diet of the Pb-exposed group ameliorated Pb deteriorating changes. This positive effect might be correlated with immunostimulant and antioxidant effects of βG and MOS as confirmed previously by Selim, Reda^71^.

Treatment of Pb group within dietary MOS and βG ameliorated the Pb progressive alterations. At the level of electronic microscope, dietary βG and MOS did not affect the tissues of Nile tilapia, which persisted in health status as control group. This positive effect of MOS and βG in hepatic tissues might be due to their roles as immune and antioxidant inducers as evidenced by promotion of liver SOD and GSH beside lowered MDA content, these positive effect reported previously by Abu-Elala et al.^78^.

The maximum permissible level of Pb residues in fish tissues which suggested by the Food and Agriculture Organization (FAO) was 0.5 ppm (FAO, 2003)^79^. The present data showed a significant elevation in Pb bioaccumulation in the tissues of gills, muscles, and livers of Pb-exposed tilapias after 8 weeks. The Pb accumulation level in the selected tissues in all studied groups were in the following order; liver > gills > muscles, and these results were in harmony with the data recorded by Cogun et al.^80^ who detected analogous distribution outline of Cd and Cu in tissues of Nile tilapia. The detected Pb residues herein exceeded the recommended level by FAO and might cause a hazard to human health. Pb primarily entered the fish body through gills, then passes to blood stream via gill membrane and deposited in various tissues according to the physiological and biological roles of each organ^23^. From a food safety perspective, while MOS/βG supplementation reduced muscle Pb by 42%, residues in the Pb + MOS/βG group (1.24 mg kg^−1^) remained 2.5-fold above the FAO/WHO maximum permissible limit of 0.5 mg kg^−1^ for fish muscle^81^. This indicates that dietary interventions alone cannot fully protect human consumers when fish are exposed to high waterborne Pb concentrations (10 mg L^−1^). Therefore, MOS/βG supplementation should be considered as part of an integrated approach combining source control, water treatment, and reduced exposure duration rather than as a standalone solution for heavily contaminated aquaculture systems. In moderately polluted environments (Pb < 2 mg L^−1^), MOS/βG may provide adequate protection to maintain muscle Pb below regulatory thresholds.

The liver in the present study demonstrated a greater propensity to accumulate Pb compared to the gills and muscles. This might be related with the crucial role of liver in metabolism, detoxification and redistribution of metals^28^. The present data were in accordance with Chi et al.^82^ who indicated that the active metabolic organs as the liver gathers more amounts of metals than other organs or tissues. Previous studies on several fish species indicated that the liver serves as the chief organ for accumulation of metals^83^. Other study were detected high levels of Pb accumulation in liver of juvenile rockfish *Sebastes schlegelii* after 4 weeks exposure to different doses of Pb (0, 120, and 240 mg/L) on diet^84^.

The gills’ properties as high permeability and direct contact with environmental pollutants had made this organ to be the chief goal organ for metal accretion, before circulating the metal to the fish tissues through bloodstream^85^. On contrary, the gills of Nile tilapia in the present study considered a temporary target organ of Pb accumulation. Due to their continuous interaction with the surrounding environment and their involvement in both respiratory and excretory processes, gills are directly impacted by environmental contaminants^86^. In the current study, the lowest levels of Pb found in the gills, as contrasting to the liver, may be ascribed to the reduced binding affinity of Pb on the gill surface. Additionally, this observation could be linked to the advance of protective mechanisms, like increased mucous secretion in the gills, which functions as a protective ion trap^87^. In contrast to our results, previous studies revealed that high levels of Pb precipitation was noticed in the gill tissues, owing to direct intact within metal ions in the water during osmoregulation and respiration^88^.

The lowest bioaccumulation rate of Pb in muscles in current study was interrelated with the lower metabolic activity, as the muscular tissues not active sites for detoxification and transportation of metals as confirmed by Uluturhan, Kucuksezgin^89^. In accordance with the present study, Dural et al.^90^ studied Pb accumulation in muscles of three fish species. They found significant lower Pb residue in muscles than gills and liver.

Dietary MOS and βG supplemented to Pb group lowered the Pb residues in the tested tissues. The reduction of Pb residues in tilapia tissues fed on MOS and βG might be due to the potential of MOS and βG to accelerate of Pb excretion through the urine rather than deposition. Our result were in agreement with Ayyat et al.^42^ confirmed that MOS tend to accumulate lower concentrations of Pb in the bodies of Pb-intoxicated fish. The inhibitory effects of dietary βG and MOS on Pb absorption and accumulation remain unassessed and necessitate further investigation. The mechanisms underlying MOS/βG-mediated Pb reduction require further investigation. Future studies should quantify antioxidant enzyme activities (SOD, CAT, GPx), lipid peroxidation (MDA), metallothionein expression, fecal/urinary Pb excretion, and metal transporter gene expression (e.g., DMT1, ZIP transporters) to elucidate the protective pathways.

## Conclusion

This study demonstrates that dietary supplementation with 0.3% mannan oligosaccharides (MOS) and β-glucan effectively mitigates Pb-induced toxicity in Nile tilapia by restoring serum protein balance, reducing hepatic enzyme leakage, preserving tissue and cellular integrity, and limiting Pb bioaccumulation in edible muscles, liver, and gills. These findings validate the cytoprotective and detoxification-enhancing properties of yeast-derived prebiotics under environmentally relevant contamination levels. From a practical standpoint, MOS/βG inclusion at low dietary levels is commercially feasible due to its wide availability, low cost, and established use as a functional feed additive in intensive aquaculture. Therefore, integrating MOS/βG into feeding programs could enhance fish robustness and reduce losses in Pb-polluted regions while improving the safety of aquaculture-derived food products. However, the present study used only a single Pb concentration and a single dose of MOS/βG, and oxidative stress and immune gene markers were not assessed. Future studies incorporating dose–response designs, multiple pollutant stressors, and mechanistic biomarker analyses (e.g., antioxidant enzymes, inflammatory gene expression) will strengthen the understanding of how yeast-derived prebiotics enhance metal resilience. Long-term farm-scale trials are also recommended to evaluate economic profitability and large-scale applicability.

## Supplementary Information

Below is the link to the electronic supplementary material.

Supplementary Material 1

## Data Availability

The authors confirm that the data supporting this study’s findings are available within the article.
