# Supplementary material for: Yeast prebiotics mitigate lead toxicity in Nile tilapia through physiological and ultrastructural improvements
Source: Sci Rep. 2026 Mar 5;16:8273. doi: 10.1038/s41598-026-37841-z (PMC12966497; doi:10.1038/s41598-026-37841-z)
Supplement: Supplementary file 1 — Supplementary Material 1 [file 41598_2026_37841_MOESM1_ESM.docx]

Supplementary Table S1: Complete ANOVA Statistics

| Parameter | F-value | df (between, within) | Exact p-value | Post-hoc significant comparisons (Tukey HSD) |
| --- | --- | --- | --- | --- |
| Serum Biochemical Parameters |  |  |  |  |
| Total protein (g/dL) | 45.32 | 3, 176 | < 0.001 | Pb < Control = Pb+MOS < MOS |
| Albumin (g/dL) | 32.18 | 3, 176 | < 0.001 | Pb < Control = MOS = Pb+MOS |
| Globulin (g/dL) | 28.64 | 3, 176 | < 0.001 | Pb < Control < MOS = Pb+MOS |
| ALT (U/L) | 68.47 | 3, 176 | < 0.001 | MOS < Control = Pb+MOS < Pb |
| AST (U/L) | 52.19 | 3, 176 | < 0.001 | MOS < Control = Pb+MOS < Pb |
| ALP (U/L) | 89.64 | 3, 176 | < 0.001 | MOS < Control = Pb+MOS < Pb |
| Pb Bioaccumulation (mg/g wet weight) |  |  |  |  |
| Muscle Pb | 78.23 | 3, 32 | < 0.001 | Control = MOS < Pb+MOS < Pb |
| Gill Pb | 124.56 | 3, 32 | < 0.001 | MOS = Control < Pb+MOS < Pb |
| Liver Pb | 156.89 | 3, 32 | < 0.001 | MOS = Control < Pb+MOS < Pb |

Note: Data analyzed by one-way ANOVA followed by Tukey's multiple comparison test. df = degrees of freedom (between groups, within groups). n = 45 fish per treatment for serum parameters; n = 9 tissue samples per treatment for Pb bioaccumulation. Significance level α = 0.05.​
